# Meningitis caused by *Rhodotorula mucilaginosa* in human immunodeficiency virus seropositive patient

**DOI:** 10.4103/0972-2327.44561

**Published:** 2008

**Authors:** V. P. Baradkar, S. Kumar

**Affiliations:** Department of Microbiology, Lokmanya Tilak Municipal Medical College and General Hospital, Sion, Mumbai, India

**Keywords:** Human immunodeficiency virus, meningitis, *Rhodotorula*

## Abstract

*Rhodotorula* species may be responsible for systemic infection in immunocompromised patients. Meningitis by *Rhodotorula* species in human immunodeficiency virus (HIV) infected persons has been reported previously. We report a case of meningitis caused by *Rhodotorula mucilaginosa* in a 36-year-old HIV seropositive male patient who presented with fever, altered sensorium and features of meningeal irritation i.e. neck rigidity. The Cerebrospinal fluid (CSF) cell counts were high, showing 150 cells/mm^3^, with 60% lymphocytes and 40% polymorphs, and protein content of 100 mg%; glucose was 60 mg%. The diagnosis was confirmed by culture on Sabouraud's Dextrose agar. The patient was treated successfully with intensive Amphotericin B (1 mg/kg), for two weeks, followed by oral Itraconazole (400 mg daily), for a period of two months. The patient was started on anti retroviral therapy. He did not show any relapse of the symptoms when the last follow up was done six months after the date of discharge.

## Introduction

Invasive fungal disease continues to be a significant problem among immunocompromised patients. Most fungal infections are caused by commonly recognized opportunistic fungi such as *Aspergillus* species, *Candida* species, *Cryptococcus neoformans* and dimorphic fungi.[1] Fungi like *Trichosporon beigelii*, Malassezia furfur, Geotrichum candidum and Rhodotorula species are emerging pathogens of immunocompromised patients.[1–3] Rhodotorula species has been reported to cause septicemia, sepsis, meningitis, endocarditis, keratitis, ventriculitis, peritonitis, central venous catheter infection and endophthalmitis.[3–10] We report a case of meningitis caused by Rhodotorula mucilaginosa in an HIV infected patient.

## Case History

A 36-year-old male patient presented with a history of headache and fever for the past seven days, and altered sensorium since the last three days. On enquiry, it was found that he had had pulmonary tuberculosis two years back, for which he had received anti tubercular therapy comprising of 2H_3_ R_3_ Z_3_ E_3_ (H: Isoniazid (600 mg), R: Rifampicin (450 mg), Z: Pyrazinamide (1500 mg), Ethambutol (1200 mg) for two months, followed by 4H_ 3_ R_3_ (H: Isoniazid (600 mg), R: Rifampicin (450 mg), for a period of four months.

On general examination the patient was found to be febrile (100° F); there was no pallor, icterus or lymphadenopathy noted.

On neurological examination, the patient was found to have altered sensorium and neck rigidity. Other motor and sensory examinations were unremarkable. The cardiovascular and respiratory system examinations were within normal limits. Abdominal examination per se did not reveal any guarding, rigidity, free fluid or organomegaly.

The patient's laboratory investigations revealed hemoglobin (Hb) of 12 gm%, erythrocyte sedimentation rate (ESR) of 8 mm/hr, total leukocyte count (TLC) - 7600/mm^ 3^, with lymphocytes 60% and polymorphs 40%. The liver and kidney functions were within normal limits. An X-ray of the chest did not reveal any abnormality. Serological testing for HIV antibodies was performed using Enzyme linked immunosorbent assay (ELISA), which revealed that he was reactive to HIV 1 antibodies. The CD_4_ counts were 196 cells/mm^3^; however, viral load and computerized tomography (CT) scan of the brain was not performed.

**Figure 1:**
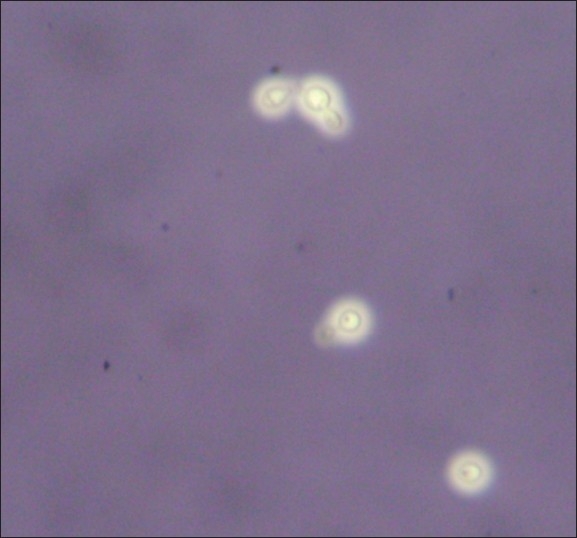
10% Nigrosin mount (X400) showing capsulated yeasts

**Figure 2:**
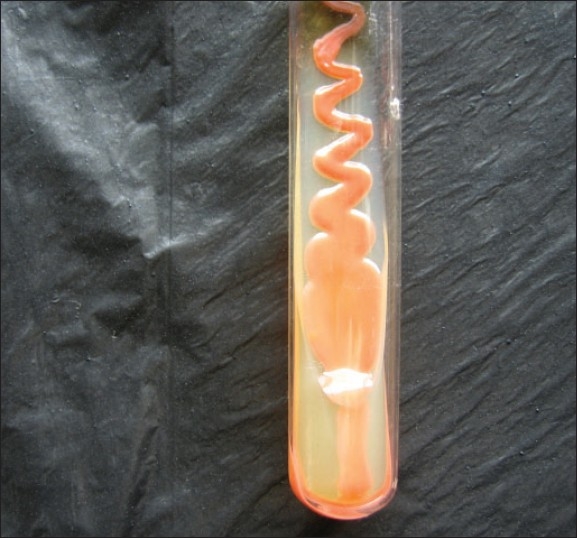
Red mucoid colonies of *Rhodotorula* species on Sabouraud's dextrose agar

In view of the altered sensorium and neck rigidity, a lumbar puncture was performed. The cerebrospinal fluid (CSF) was examined by microscopy, with 10% Nigrosin staining, Gram staining, and Ziehl-Neelsen staining, and bacteriological and fungal culture was done. The CSF cell counts were high, showing 150 cells/mm^3^, with 60% lymphocytes & 40% polymorphs, protein content of 100 mg%; glucose was 60 mg%. Nigrosin staining showed encapsulated budding yeast cells [Fig. 1]. Gram stained smear showed Gram positive budding yeast cells. Initially, presumptive diagnosis of cryptococcal meningitis was made on the basis of Nigrosin mount and Gram staining. Ziehl-Neelsen staining was negative for acid fast bacilli. No organisms grew on bacteriological culture of CSF. A red pasty mucoid growth [Fig. 2] was seen on CSF culture on Sabouraud's Dextrose agar with antibiotics (Chloramphenicol 0.05 mg/L, cycloheximide 0.5 mg/L) and without antibiotics at 25°C and 37°C. Gram staining from colony revealed Gram positive budding yeast cells. These findings, along with the biochemical tests[11] and the absence of ballistospore formation, helped us to diagnose the pathogen as Rhodotorula mucilaginosa and the meningitis as a case of Rhodotorula meningitis. The possibility of contamination was ruled out by repeated isolation from another CSF sample of the patient, collected aseptically, two days after the initial report of capsulated yeasts seen in the CSF was given. The blood cultures of the patient, however, showed no growth in culture.

The patient was administered intravenous Amphotericin B (1 mg/kg/day) for two weeks. He responded well to the treatment, with the fever subsiding after one week of Amphotericin B therapy; he gradually regained consciousness. The patient was later started on oral Itraconazole (400 mg daily) for a period of two months. The patient was discharged after a period of four weeks from the date of admission and was started on anti retroviral therapy namely Zidovudine (300 mg) + Lamivudine (150 mg) + Nevirapine (200 mg), because of his low CD_4_ counts. The patient had not shown any relapse of symptoms when the last follow up was done six months after the date of discharge.

## Discussion

The incidence of fungal infections of Central nervous system (CNS) has shown a steep rise, largely due to the advent of acquired immune deficiency syndrome (AIDS), and the widespread use of broad spectrum antibiotics, steroids and immunosuppressive drugs.[4] Rhodotorula species is emerging in immune compromised patients, especially in HIV infected patients.[1–3] Rhodotorula species is common saprophytes in the environment. However, their etiological role in immunocompromised patients has been described only rarely.[1–3]

Rhodotorula fungal infections of the CNS continue to be uncommon. Rhodotorula belongs to the family Sporidiobolaceae and Phylum Basidiomycota.[11] It produces mucoid yeast like soft, pasty colonies with red pigment on Sabouraud's Dextrose agar (SDA) medium. Microscopically, the unicellular cells of this fungus are spherical in shape, size varying from 4 mm to 6 mm, surrounded by a capsule. No ascospores are present. Many species of Rhodotorula have been described, but Rhodotorula mucilaginosa is the commonest species isolated from clinical specimens.[1–3] Rhodotorula species and Cryptococcus neoformans have many similarities and both produce meningitis. Hence each may be mistaken for the other. Rhodotorula species differs from Cryptococcus neoformans in its carotenoid pigments.[1–4] Its isolation from normally sterile sites such as blood and CSF is of greater significance.[5 Rhodotorula species has been reported to cause septicemia, sepsis, meningitis, endocarditis, keratitis, ventriculitis, peritonitis, central venous catheter infection and endophthalmitis.[3,4,6,10,12,13] Pore and Chen[14] reported a case of Rhodotorula mucilaginosa meningitis in patients with acute lymphoblastic leukemia. He detected encapsulated budding yeast cells and confirmed it by culture.

A case of meningitis in HIV infected persons was reported by Gyaurgieva *et al*[6] Recently Thakur et al.[4] described Rhodotorula mucaliginosa meningitis in an HIV infected patient from Himachal Pradesh, India. The patient was initially on intravenous Amphotericin B; later, intravenous Flucytosine (25 mg/kg) was added. However, in spite of the treatment, the patient died. On the other hand, our patient responded to intravenous Amphotericin B treatment. A study of the susceptibility profile of 29 clinical isolates of Rhodotorula spp. revealed that Fluconazole, itraconazole and voriconazole were inactive in vitro against a majority of Rhodotorula isolates, while Amphotericin B and flucytosine exhibit good activity, being reasonable alternatives for empirical treatment.[15] The new and investigational triazoles all have some in vitro activity, with ravuconazole being the most active.[10] Treatment of Rhodotorula infection involved the removal of central venous catheters if any, and, generally, amphotericin B or fluconazole therapy for 14 days.[11]

The recognition of unusual yeasts as agents of occasional life-threatening infections and their unpredictable antifungal susceptibilities increases the burden on the clinical microbiology laboratory to complete species identification and determine minimal inhibitory concentration (MIC).

To conclude, Rhodotorula species should be kept in mind while diagnosing and treating cases of meningitis, especially in HIV infected patients.
